# Orange Pomace Improves Postprandial Glycemic Responses: An Acute, Randomized, Placebo-Controlled, Double-Blind, Crossover Trial in Overweight Men

**DOI:** 10.3390/nu9020130

**Published:** 2017-02-13

**Authors:** C.-Y. Oliver Chen, Helen Rasmussen, Alison Kamil, Peng Du, Jeffrey B. Blumberg

**Affiliations:** 1 Antioxidants Research Laboratory, Jean Mayer USDA Human Nutrition Research Center on Aging, Tufts University, Boston, MA 02111, USA; helen.rasmussen@tufts.edu (H.R.); alisonk730@gmail.com (A.K.); dupengfm@163.com (P.D.); jeffrey.blumberg@tufts.edu (J.B.B.); 2 The Institute of Aviation Medicine, Airforce, Beijing 100142, China

**Keywords:** glucose, insulin, men, orange pomace, postprandial

## Abstract

Orange pomace (OP), a fiber-rich byproduct of juice production, has the potential for being formulated into a variety of food products. We hypothesized that OP would diminish postprandial glycemic responses to a high carbohydrate/fat breakfast and lunch. We conducted an acute, randomized, placebo-controlled, double blind, crossover trial with 34 overweight men who consumed either a 255 g placebo (PLA), a low (35% OP (LOP)), or a high (77% (HOP)) dose OP beverage with breakfast. Blood was collected at 0, 10, 20, 30, and 45 min and at 1, 1.5, 2, 3, 4, 5, 5.5, 6, 6.5, 7, and 8 h. Lunch was consumed after the 5.5-h blood draw. OP delayed the time (T_max_1) to the maximum concentration (C_max_1) of serum glucose during the 2-h period post breakfast by ≥36% from 33 (PLA) to 45 (HOP) and 47 (LOP) min (*p* = 0.055 and 0.013, respectively). OP decreased post-breakfast insulin C_max_1 by ≥10% and LOP delayed the T_max_1 by 14 min, compared to PLA at 46 min (*p* ≤ 0.05). HOP reduced the first 2-h insulin area under concentration time curve (AUC) by 23% compared to PLA. Thus, OP diminishes postprandial glycemic responses to a high carbohydrate/fat breakfast and the second meal in overweight men.

## 1. Introduction

Observational evidence suggests that diets rich in plant-based foods are associated with a reduced risk of a number of chronic diseases, e.g., cardiovascular disease and type 2 diabetes mellitus (T2DM) [1,2,3]. These beneficial correlations can be partly attributed to the bioactive constituents in plant-based foods, including vitamins, minerals, phytochemicals, and fiber [4]. Of these compounds, dietary fiber has been found to improve total and low density lipoprotein (LDL) cholesterol and blood pressure and reduce postprandial glycemia in intervention studies [5,6,7,8]. These beneficial effects are particularly pertinent for people following dietary recommendations to manage glucose intolerance and T2DM [9,10].

Dietary fiber includes complex carbohydrates that are non-digestible in the upper gastrointestinal (GI) tract. Since mammals do not produce enzymes capable of hydrolyzing fiber into its constituent monomers, undigested fiber travels intact through the GI tract to the colon, where it is available for fermentation by resident microbiota [11]. Dietary fiber is commonly classified according to its solubility in water, i.e., into insoluble (IDF) and soluble dietary fiber (SDF). In general, fiber that is less soluble (cellulose, hemi-cellulose) is linked to improved laxation, and those that are more soluble, such as β-glucan and pectin, are associated with lower blood lipids and attenuated postprandial hyperglycemia [12]. In addition, the viscosity and fermentability of dietary fiber can mediate some of these responses. Viscous fiber increases the viscosity of the meal bolus, which delays nutrient absorption, and fermentable dietary fiber increases the production of short chain fatty acids (SCFA) [13]. SCFA, particularly butyric and propionic acids, are recognized as beneficial to human health via a number of mechanisms, e.g., facilitating the production and secretion of peptide YY (PYY) and glucagon like peptide-1 (GLP-1), two endocrine factors capable of mediating insulin secretion and sensitivity [14].

During orange juice production, orange pomace is produced. It is a fiber-rich waste byproduct, which also contains an edible portion of the fruit that includes segments, broken pulp sacs, and a center core. Orange pomace is not fully utilized as an ingredient providing added value to the processed foods. In addition to fiber, orange flavanones entrapped in the fiber are also lost during juice production and may help regulate the postprandial glucose response [15,16,17]. Thus, we examined the effect of a beverage containing orange pomace and water on blood glucose and insulin responses to a standard high fat and high carbohydrate breakfast in a randomized, double blind, placebo controlled, crossover trial in overweight men.

## 2. Experimental Section

### 2.1. Subjects

Men aged 30–65 years with a body mass index (BMI) between 25 and 29.9 kg/m^2^ were recruited from the Boston metropolitan area. Interested subjects were screened initially by telephone, which was then followed by an on-site interview. All subjects signed written informed consent forms before any study procedures were performed. The exclusion criteria included T2DM (fasting glucose > 7 mmol/L), endocrine disorders, a myocardial infarction/stroke in the past 12 months, renal or bowel disease, or a history of cholestatic liver or pancreatitis. Also excluded were those on drug treatment for hyperlipidemia, hypertension, inflammation or hypercoagulation, alcoholics, those planning or being on a weight reduction regimen, smokers, and hematology markers and clinical chemistry profiles outside acceptable ranges (e.g., creatinine >1.5 mg/dL, calcium 8.3–10.2 mg/dL, phosphorous 2.3–4.7 mg/dL, alanine aminotransferase (ALT) >55.5 U/L, aspartate aminotransferase (AST) >51.0 U/L, total bilirubin >1.5 mg/dL, triglycerides ≥300 mg/dL). The final eligibility of subjects was confirmed by the study physician. During the on-site screening visit, blood was collected for the analysis of hematology markers and a clinical chemistry profile. Further exclusion criteria were regular use (>2 times/week) of any acid-lowering medications, laxatives or anti-diarrheal medications, systolic blood pressure > 150 mmHg and/or diastolic blood pressure >95 mmHg, any cardiovascular diseases (CVD), GI diseases/issues or medications influencing GI absorption, renal or chronic kidney disease, rheumatologic diseases, active treatment for cancer of any type ≤1 year, regular use of oral steroids except topical over-the-counter steroids, regular use of any dietary supplements containing vitamins, minerals, herbal or other botanical preparations, fish oil (including cod liver oil) or homeopathic remedies, usual weekly ethanol intake ≥2 drinks, and an infrequent (<3/week) or excessive (>3/day) number of regular bowel movements. The conduct of all work performed in the trial was first approved by the Institutional Review Board of Tufts Medical Center/Tufts University. The study was registered in the clinicaltrials.gov (registration number: NCT02112851). The study was approved by the Institutional Review Board of Tufts Medical Center/Tufts University (approval number: 11067).

### 2.2. Study Design

The trial was conducted in the Clinical and Translational Research Center (CTRC) of the Tufts Translational and Clinical Science Institute (CTSI), Boston, MA, USA. The trial was an acute, randomized, placebo controlled, double blind, 3-way crossover study. Although neither the investigators nor the subjects were aware of which treatments the subjects received, the difference in viscosity between the three test beverages might have been evident to the participant when the beverages were consumed via a black straw. The beverages were packaged in a metal bottle. The investigators and those who performed sample and data analyses were completely blind. Eligible subjects were randomly assigned to receive one of three test beverages, 240 mL (255 g) placebo (PLA), low dose orange pomace (LOP), and high dose orange pomace (HOP), along with the standard breakfast. All subjects consumed the breakfast with the test beverage within 15 min. No other food or beverages were provided at this time. After 30 min, subjects could consume up to 300 mL water within the first 3 h. The randomization schematics were prepared by a biostatistician using a standardized computer program for the three treatment groups in a cross-over design to ensure that each treatment was followed by a different treatment in one of 6 possible sequences. A fasting blood sample was first taken at baseline. After the completion of the breakfast and assigned beverage, 11 blood samples were drawn until 5.5 h from the end of the breakfast when a medium fat lunch was given to each subject, followed by the collection of four additional blood samples. Thus, a total of 16 blood samples (baseline, 10 min, 20 min, 30 min, 45 min, and 1, 1.5, 2, 3, 4, 5, 5.5, 6, 6.5, 7, and 8 h) were collected from an indwelling catheter or syringe in one of the arms of each subject. The 10, 20, and 45 min time points were added to postprandial glucose and insulin kinetics with an intention to obtain the maximum glucose (insulin) concentration (C_max_) and the time to reach the C_max_ (T_max_). A washout period (≥2 weeks) was implemented between test beverages. Two days prior to each intervention visit, subjects were instructed to follow a low phytochemical diet. On the day prior to the intervention visits, they consumed low phytochemical breakfast, lunch, and dinner meals prepared by the study to ensure the compliance of a flavonoid-free period. The foods provided included plain bagels, cream cheese, sliced turkey, bread, butter, macaroni and cheese, pretzels, chicken, buttered eggs (yolkless), noodles with parmesan cheese, saltine crackers, and angel food cake. A low phytochemical lunch was provided to the subjects on the day of receiving a test beverage. A 24-h dietary recall record was collected on the day before the intervention visits to confirm their compliance to the low phytochemical diet.

### 2.3. Study Beverages and Meals

The OP added to the LOP and HOP test beverages was comprised of the micronized edible part of the whole orange, which is a waste by-product generated during the production of Tropicana Pure Premium Orange Juice™. To obtain OP, oranges were harvested, transported, inspected, cleaned, and graded using standard industry and good manufacturing practices. The OP is rich in fiber (40:60 ratio of soluble to insoluble) and contains small amounts of micronutrients, and a high proportion of the flavonoids found in the whole orange. The same OP has been tested by others for its effect on glucoregulation and cognitive performance in humans [18,19]. The LOP and HOP were prepared to contain 35% and 77% (weight/weight) of OP and 64.6% and 22.5% water, respectively. The minor ingredients included citric acid (0.38%), stevia as a sweetener (0.006%), and natural orange flavor (0.054%). The PLA beverage was comprised of mineral water, whey protein, stevia for sweetness, β-carotene for color, and symrise orange aroma for orange flavor. The beverages were closely matched for energy (31.6 kcal), protein (1.79 g), and carbohydrate (6.12 g) content. The fiber content of PLA, LOP, and HOP was 0, 2.55, and 5.48 g, respectively. The fiber content of the HOP is equivalent to that typically consumed when eating 250 g (1.6 servings) of the edible portion of an orange per US Labelling Standards. LOP and HOP contained approximately 127 and 272 mg flavonoids, principally hesperidin at 81% [18]. All ingredients for the test beverages, except water, were provided by PepsiCo Inc. (Purchase, NY, USA) and kept frozen (−20 °C) until use. Test beverages were prepared within 24 h prior to consumption. Nutrient information of the foods consumed with the test beverages is presented in Table 1. The total carbohydrate and energy intake in the breakfast was 70.12 g and 808.6 kcal, respectively.

### 2.4. Determination of Serum Glucose and Insulin

Whole blood was allowed to clot at room temperature for 20 min and the serum was separated by centrifugation at 1100× *g* for 20 min at 4 °C, aliquoted, and stored at −80 °C until analysis. Glucose was measured using an enzymatic method (Olympus America, Inc., Center Valley, PA, USA). The inter-day coefficient of variation (CV) for glucose determination was 2%. Insulin was measured using a human insulin-specific RIA kit (EMD Millipore, Billerica, MA, USA). The inter-day CV for insulin determination was 5%.

### 2.5. Statistical Analysis

Values are expressed as mean ± SEM. A sample size of 33 was obtained from a power calculation using preliminary data. The effect size (difference in maximum serum glucose concentration of the first 2 h) and standard deviation for the calculation were 0.4807 and 0.9455, respectively. The sample size that gives 80% power at the 0.05 level of significance for our primary outcome that HOP would attenuate the first 2-h postprandial glucose increase after breakfast, as compared to PLA, was 33. The results are expressed as mean ± SEM. The effect of test beverages on serum glucose and insulin was assessed using a general linear mixed effects model. In the SAS PROC MIXED procedure, the independent fixed factors were sequence, period, and product, and baseline glucose or insulin value was added as a covariate. Evaluation time points (10, 20, 30, and 45 min and 1, 1.5, 2, 3, 4, 5, 5.5, 6, 6.5, 7, and 8 h) were the repeating factor, and subjects within the sequence were a random factor. Comparison between the 3 test beverages was performed using Tukey’s HSD multi-comparison test. The area under the curve (AUC) of blood glucose and insulin was calculated using the original values without being adjusted with the corresponding baseline value and the trapezoidal rule. Due to the biphasic nature of the glucose and insulin responses to sequential meal ingestion, the maximum observed glucose (insulin) concentration (C_max_) after consumption of breakfast (C_max_1, from 0 to 5.5 h) and lunch (C_max_2, from 5.5 to 8 h) and the time to reach the C_max_1 and 2 (T_max_1 and 2) were manually obtained from the kinetic curves of the serum glucose and insulin of all three interventions. The treatment effect on AUC, insulin AUC/glucose, AUC ratio, C_max_, T_max_ and the slope of glucose or insulin reduction from T_max_1 to 5.5 h was assessed using the same statistical test without the repeated measures. All analyses were performed in SAS version 9.3.

## 3. Results

**Figure 1:**
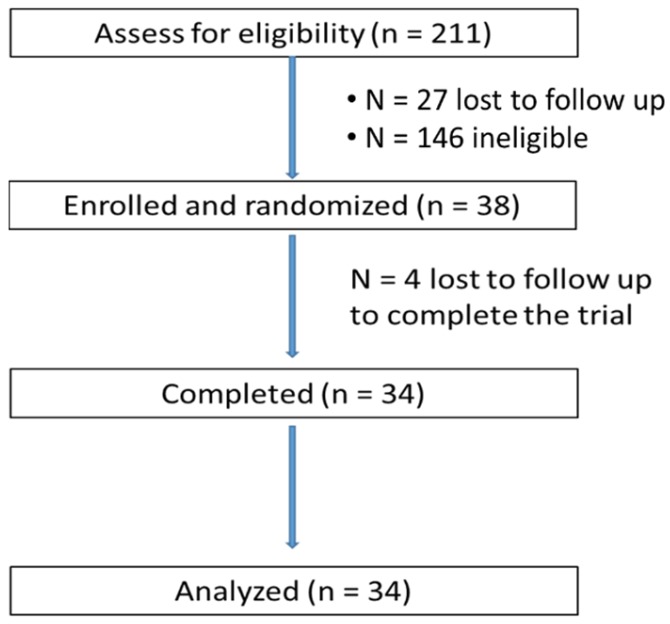
CONSORT flow diagram of the progress through the phases of a randomized, 3-way crossover, pharmacokinetics trial.

A total of 211 volunteers were assessed for their eligibility, and 38 were eligible and enrolled in the trial (Figure 1). Thirty-four subjects completed all three interventions, one subject completed two interventions and did not return for the final intervention, and three subjects were lost to follow-up after they signed the main study consent form. The characteristics of the completed subjects are presented in Table 2. The mean age of 34 completers was 52.1 ± 7.7 years, and the mean serum glucose at the screening was 91.1 ± 7.1 mg/dL. Mean body weight and BMI did not change during the course of the study. The amount of water consumed during the three intervention visits did not differ. No apparent adverse effects related to consumption of the study beverages were noted.

**Figure 2:**
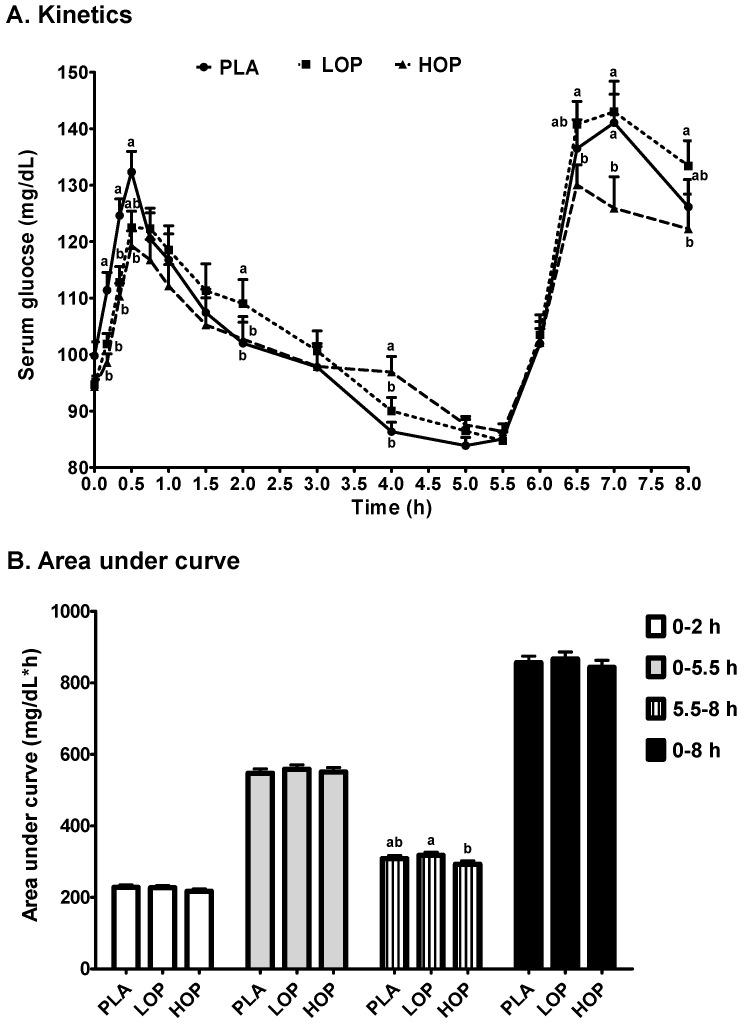
(**A**) Kinetics and (**B**) area under the curve of serum glucose in the subjects consuming one of three study beverages, placebo (PLA), Low orange pomace (LOP), and High orange pomace (HOP), along with a high fat and carbohydrate breakfast and a high fat and carbohydrate lunch after the 5.5-h blood draw. Serum glucose was measured at 0, 10, 20, 30, and 45 min and 1, 1.5, 2, 3, 4, 5, 5.5, 6, 6.5, 7, and 8 h. The area under the curve of serum glucose was calculated in four time periods; 0–2 h, 0–5.5 h, 5.5–8 h, and 0–8 h. ^a,b^ Means at the same time point without sharing the same letter differ, tested by the general linear mixed effects model followed by the Tukey HSD multi-comparison test, *p* ≤ 0.05.

Serum glucose values at the baseline of each intervention were comparable, ranging from 94.3 mg/dL for LOP to 99.8 mg/dL for PLA (Figure 2A). OP delayed the time (T_max_1) to the maximum concentration (C_max_1) of serum glucose during the first 5.5-h period post breakfast by ≥36% from 33 (PLA) to 45 (HOP) and 47 (LOP) min (*p* = 0.055 and 0.013, respectively) but did not affect the C_max_1 (Table 3). The T_max_2 during the last 2.5 h-period post lunch of two OP doses was not different from that of PLA. Interestingly, it was noted that the T_max_2 of HOP was 9.2% shorter than that of LOP (*p* ≤ 0.05). When comparing serum glucose values at each time point between the three study beverages, the impact of OP, particularly HOP, on glucose excursion was statistically significant (Figure 2A). The lower serum glucose concentration at 10, 20, and 30 min post breakfast and the higher level at 4 h were noted in the subjects consuming HOP as compared to PLA. The LOP beverage only diminished the breakfast-induced increase in serum glucose at 10, 20, and 30 min. The effect of OP on glucose excursion persisted to the lunch, but only HOP significantly decreased the serum glucose levels by ≥10.7%, as compared to PLA. OP did not modify the overall AUC of serum glucose, although HOP significantly attenuated glucose concentrations at most time points (*p* ≤ 0.05) (Figure 2B). The slope of the serum glucose decrease from the T_max_1 to 5.5 h post breakfast was 25.8% smaller for HOP than LOP and PLA (*p* ≤ 0.05), and LOP did not display the same impact (Table 3).

**Figure 3:**
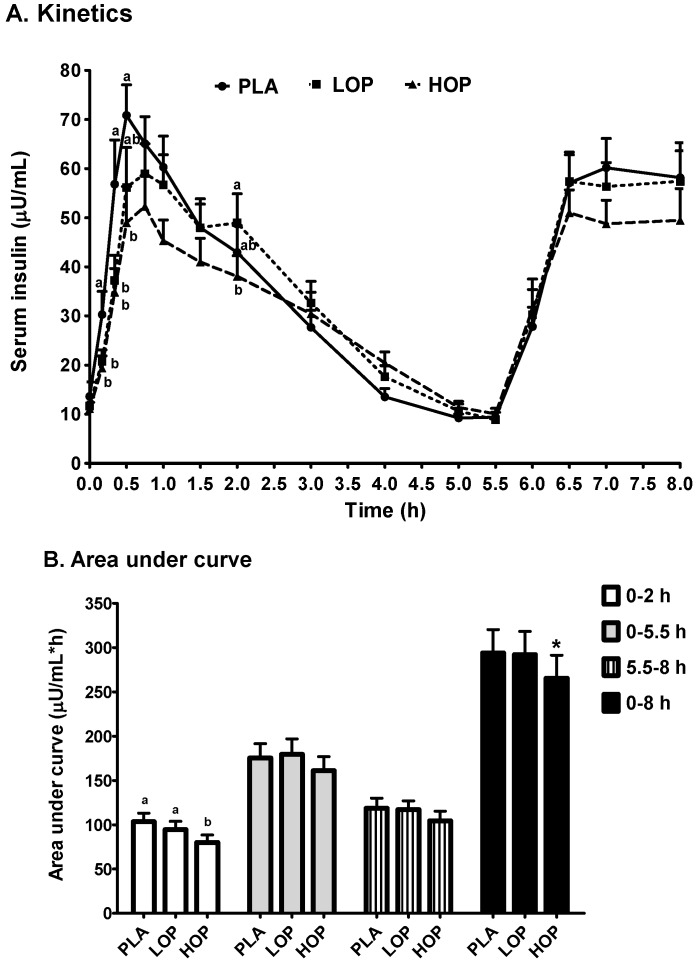
(**A**) Kinetics and (**B**) area under the curve of serum insulin in the subjects consuming one of three study beverages, placebo (PL), Low (LOP), and High (HOP) OP, along with a high fat, carbohydrate breakfast and a high fat, carbohydrate lunch after the 5.5-h blood draw. ^a,b^ Means at the same time point without sharing the same letter differ, tested by the general linear mixed effects model, followed by the Tukey HSD multi-comparison test, *p* ≤ 0.05. * Means at the same period tend to differ from the other 2, *p* ≤ 0.1.

Serum insulin values at the baseline did not differ between the three study beverages (Figure 3A). OP decreased the post-breakfast insulin C_max_1 by ≥10%. LOP delayed the T_max_1 by 14 min compared to PLA at 46 min (*p* ≤ 0.05), and HOP tended to have the same effect (Table 3). However, OP did not affect the C_max_2 and T_max_2. When the serum insulin values of each time point were compared between the three study beverages, the impact of OP, particularly HOP, on postprandial insulin concentrations was statistically significant (Figure 3A). At 10 and 20 min post breakfast, both HOP and LOP significantly suppressed the insulin values by ≥31.6%, as compared to PLA. The suppression continued to 30 min post breakfast in subjects consuming HOP. HOP reduced the first 2-h insulin AUC by 23% compared to PLA and tended to decrease the overall AUC, as well as that of the first 5.5-h period (Figure 3B). Unlike the noted effect on the post-lunch serum glucose values, OP did not affect the magnitude of the lunch-induced insulin surge. The slope of the serum insulin decrease from the T_max_1 of each individual during each intervention visit to 5.5 h post breakfast was 24.2% less steep after HOP than LOP and PLA (*p* ≤ 0.05), while there was no difference between LOP and PLA (Table 3).

**Figure 4:**
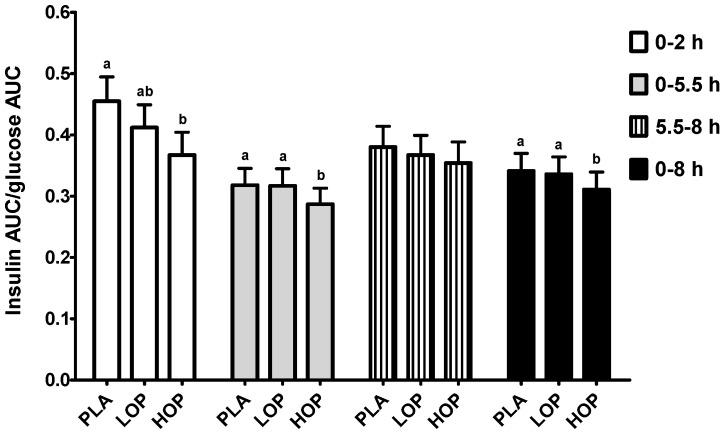
The ratio of the serum insulin area under the concentration-time curve (AUC) to the serum glucose AUC in the subjects consuming one of three study beverages, placebo (PL), Low (LOP), and High (HOP), along with a high fat and carbohydrate breakfast and a high fat and carbohydrate lunch after the 5.5-h blood draw. ^a,b^ Means at the same time point without sharing the same letter differ, tested by the general linear mixed effects model, followed by the Tukey HSD multi-comparison test, *p* ≤ 0.05.

The impact of OP on glucose-stimulated insulin secretion, defined as the insulin AUC/glucose AUC ratio, is presented in Figure 4. HOP decreased the ratio by 19%, 10%, and 9% for 0–2, 0–5.5, and 0–8 h post-treatment period, respectively, as compared to PLA (*p* ≤ 0.05). The ratio of the first 2 h after the LOP consumption was not significantly different from that of HOP and PLA, but the value of the 0–5.5 and 0–8 h periods was different from that of HOP in the same periods. The insulinogenic index (IGI) was also calculated using the formula (insulin*_t_*_=30_ − insulin*_t_*_=0_)/(glucose*_t_*_=30_ − glucose*_t_*_=0_) [20], but the values were not different between the three study beverages.

## 4. Discussion

Frequent and marked postprandial metabolic responses contribute to the development and progression of metabolic disorders, e.g., metabolic syndrome, T2DM, and CVD [21,22,23,24], mediated importantly via hyperglycemia-induced endothelial dysfunction, oxidative stress, and inflammation [22]. Thus, foods releasing their carbohydrates at a slow rate into the blood and providing sustained blood glucose levels are preferable for reducing the risk of metabolic disorders, as most people spend much of the day, e.g., up to 18 h, in a postprandial state. In this acute, crossover intervention study, we examined whether a beverage formulated with OP, a byproduct of orange juice production containing insoluble and soluble fiber and flavonoids, would modulate the postprandial glycemic response to a high fat and carbohydrate breakfast in men without diabetes or metabolic syndrome. OP attenuated the postprandial glucose and insulin responses to the co-consumed breakfast with the effect extending to lunch.

Foods consumed in mixed meals have a marked impact on the rate of carbohydrate digestion and absorption, which, in turn, dictate postprandial glucose and insulin responses [25,26,27]. Among various dietary constituents, the strongest influence on postprandial glucose and insulin kinetics is provided by the amount of digestible carbohydrates, i.e., monosaccharides (glucose, fructose), disaccharides (sucrose, lactose), and certain polysaccharides (starch), in any given consumed meal. However, factors other than the amount of carbohydrate can also have profound effects on glucose metabolism. Recently, Russell et al. [26] reviewed the evidence that carbohydrate, protein and fat, micronutrient vitamins and minerals, non-nutrient phytochemicals including fiber, and additional foods including low-calorie sweeteners, vinegar, and alcohol could all have clinically relevant effects on postprandial glycemia. Of all these dietary constituents, fiber plays a key role in managing the postprandial metabolic profile in healthy people as well as those with impaired glucose metabolisms [6,28]. In particular, fiber has been shown to alter the physical food form, structure, and viscosity of ingested foods, which in turn modulate carbohydrate digestion and absorption and help control postprandial glucose and insulin responses [29,30].

We found that the beverages containing OP attenuated the postprandial glycemic response to a co-consumed high fat and high carbohydrate breakfast. Further, such an effect continued to the second meal, also containing high fats and high carbohydrates. These results are consistent with those by Dong et al. [19], who tested a beverage containing the same OP dose as our HOP and achieved an attenuated glycemic response to a similarly high fat and high carbohydrate breakfast. Furthermore, we found that HOP decelerated serum glucose and insulin reductions from their respective C_max_, as compared to LOP and PLA. While mechanisms of action for this deceleration and its health implications warrant further examination, we speculate that fiber in OP may slow gastric emptying and thus prolong glucose digestion and absorption in the upper GI tract. Gastric emptying time is associated inversely with the postprandial glycemic response [31,32]. In addition to the fiber in OP, flavonoids may contribute to attenuating postprandial glycemic responses. For example, apple polyphenols have been found to attenuate glucose and insulin AUC during an oral glucose tolerance test in humans, probably via stimulating GLP-1 production [33,34,35]. Thus, fiber and flavonoids in OP may work to moderate the postprandial glycemic response in a synergistic/additive manner.

Glucose-induced insulin secretion, reflected by the insulin and glucose AUC ratio, implicates the efficiency of secreted insulin during the glucose excursion, as insulin is required for controlling diet-derived blood glucose spikes. However, blood glucose responses to a meal are not always proportional to insulin responses [36]. The smaller AUC ratio noted with the HOP suggests constituents in OP may potentiate insulin action during the excursion of the same overall amount of blood glucose. Potentiation may be related to factors other than fiber and flavonoids alone, e.g., the incretins.

In the 1980s, Jenkins et al. [37] proposed the ‘second-meal effect, in which slow release dietary carbohydrates in the initial meal improves glucose tolerance of the subsequent meal’. This phenomenon can be ascribed to dietary fiber, which can reduce glucose absorption and affect intestinal motility mediated by incretins that are synthesized in enterocytes induced by short chain fatty acids derived from colonic fermentation [38,39]. In this study, we observed the same effect in the serum glucose response to the second meal, when the HOP beverage was consumed with a high fat and high carbohydrate breakfast.

There are significant limitations in our study. First, the mechanism(s) mediating the observed changes in glycemic response were not directly measured and remain to be explored in future studies. There are several potential mechanisms accountable for the observed changes in plasma glucose disposition, and these are possibly attributable to the potential effects of fiber on gastric emptying rate [40], insulin sensitivity [41], and/or incretin production (e.g., GLP-1 and PYY) [38,42,43]. Second, as the impact of OP was observed in older, overweight men, the glycemic impact cannot be generalized to all populations, in particular to those with glucose intolerance and/or T2DM. Future studies are warranted to examine whether the comparable impact of OP appears in populations at increased risk for CVD.

## 5. Conclusions

Foods that promote low but sustained blood glucose levels may be considered advantageous with regard to metabolic control in generally healthy overweight men. The ratio between mono-, di-, and polysaccharides is no longer regarded as critical in relation to their impact on postprandial glycemia since protein, fat, and other nutrients are also importantly influence this response. Particularly, dietary fibers such as glucomannan, guar gum, psyllium, and β-glucan have been shown to exert acute improvements in postprandial glucose and insulin responses. This study demonstrates that the acute consumption of an OP-containing beverage containing 5.48 g fiber impacts postprandial glycemic excursion and also insulinemic responses in overweight men. Further, these benefits appear to continue to the second meal. Thus, our study suggests that the inclusion of high fiber foods in breakfast may be beneficial for controlling postprandial glucose spikes. Future studies are warranted to determine whether there are similar short-term and possibly additional long-term impacts in other study populations after consuming OP formulated into beverages or food, as well as to examine whether such effects can be duplicated in other fiber rich foods with different soluble/insoluble ratios.

## Figures and Tables

**Table 1:** Macronutrient composition of breakfast and lunch consumed with the study beverages.

	Fat (g)	Protein (g)	Carbohydrates (g)	Energy (kcal)
Breakfast				
Croissant	47	14	64	740
Butter	4	0	0	37
Total	51	14	64	777
Lunch				
2 Slices white bread	2	8.5	50	237
Soft cheese	13	3.6	0	131
Crisps	9	1.5	13	133
Shortbread biscuits	6	1.4	16	127
Total	30	15	80	628

**Table 2:** Demographics of the study participants at the baseline of each intervention visit *.

Attributes	PLA ^^^	LOP	HOP
Serum glucose (mg/dL)	99.8 ± 2.5	94.7 ± 1.4	94.3 ± 1.3
Serum insulin (µU/mL)	13.6 ± 3.0	11.7 ± 1.8	11.1 ± 1.2
Systolic blood pressure (mmHg)	124.3 ± 2.2	125.3 ± 1.9	123.9 ± 2.3
Diastolic blood pressure (mmHg)	76.0 ± 1.3	76.4 ± 1.1	76.3 ± 1.3
Heart rate (beat/min)	71.1 ± 2.3	69.5 ± 2.1	70.4 ± 2.4
Body weight (kg)	85.1 ± 1.3	85.0 ± 1.3	84.9 ± 1.4
Body mass index (kg/m^2^)	27.7 ± 0.3	27.6 ± 0.3	27.6 ± 0.3
Water consumed (mL/8 h)	404 ± 24.5	401 ± 20.9	364 ± 22.8

* Values are expressed as mean ± SEM; ^^^ Abbreviations: PLA, placebo; LOP, low dose orange pomace beverage, HOP, high dose orange pomace beverage.

**Table 3:** The maximum glucose and insulin concentrations (C_max_) in serum and the time to reach the C_max_ (T_max_) in subjects consuming a high fat, carbohydrate breakfast along with a beverage containing placebo, 35% (LOP) or 77% (HOP) orange pomace *.

	PLA ^^^	LOP	HOP
Glucose			
C_max_1 (mg/dL)	136.6 ± 4.0	132.1 ± 3.3	127.3 ± 3.0
T_max_1 (min)	33.3 ± 2.9 ^a^	47.2 ± 4.3 ^b^	45.0 ± 3.8 ^ab^
C_max_2 (mg/dL)	148.5 ± 4.8 ^ab^	153.6 ± 4.8 ^a^	139.4 ± 5.2 ^b^
T_max_2 (h)	7.0 ± 0.1	6.9 ± 0.1	6.9 ± 0.1
Slope (mg/dL/h, (T_max_1 to 5.5 h))	−7.85 ± 0.9 ^a^	−7.72 ± 0.7 ^a^	−5.73 ± 0.7 ^b^
Insulin			
C_max_1 (µU/mL)	83.5 ± 9.3 ^a^	75.0 ± 8.4 ^a^	62.9 ± 7.4 ^b^
T_max_1 (min)	46.0 ± 3.7 ^a^	59.7 ± 4.7 ^b^	57.4 ± 5.4 ^ab^
C_max_2 (µU/mL)	70.9 ± 6.5	71.7 ± 6.8	64.4 ± 6.9
T_max_2 (h)	7.1 ± 0.1	7.0 ± 0.1	7.1 ± 0.1
Slope (µU/mL/h, (T_max_1 to 5.5 h))	−12.95 ± 1.3 ^a^	−12.79 ± 1.7 ^a^	−9.70 ± 1.3 ^b^

* Values are expressed as mean ± SEM; ^^^ Abbreviations: PLA, placebo; LOP, low dose orange pomace beverage, HOP, high dose orange pomace beverage; ^a,b^ Means without sharing the same letter within the same row differ, tested by the general linear mixed effects model, followed by Tukey HSD multi-comparison test, *p* ≤ 0.05.
